# Identifying the factors affecting ‘patient engagement’ in exercise rehabilitation

**DOI:** 10.1186/s13102-022-00407-3

**Published:** 2022-02-07

**Authors:** Junsheng L. Teo, Zhen Zheng, Stephen R. Bird

**Affiliations:** School of Health and Biomedical Sciences, RMIT University, Melbourne, 3083 Australia

**Keywords:** COM-B, Exercise, Rehabilitation, Facilitators, Barriers

## Abstract

**Background:**

Despite the proven benefits of exercise rehabilitation for numerous health conditions, musculoskeletal injuries and recovery from surgery, patient adherence to such programs is reported to often be less than 35%. Increasing patient engagement therefore has the potential to improve patient health outcomes, benefiting the patient, their carers and the services that support them. The aims of this review were to identify the factors that contribute to ‘patient’ engagement in prescribed exercise rehabilitation using the COM-B (capability, opportunity, motivation-behaviour) framework of behavioural analysis.

**Methods:**

Five electronic databases (PubMed, Embase, Cochrane, Web of Science, and ClinicalTrials.gov) were searched. ‘COM-B’ was the key word searched for specifically within titles and abstracts, combined with either ‘physical activity’ OR ‘exercise’ included using the ‘AND’ operation. Records were then filtered and excluded following full-text screening based on the predetermined eligibility criteria.

**Results:**

Twenty studies were included in the review. The main COM-B themes highlighted for improving patient engagement were: capability—improving patient knowledge and cognitive skills for behavioural regulation, such as ‘action planning’ and ‘action control’, which could also benefit time-management; opportunity—a balanced life situation that enabled time to be devoted to the exercise program, social support, easily accessible and affordable resources and services; and motivation—increasing patient levels of self-efficacy and autonomous motivation, which were noted to be influenced by levels of perceived ‘capability’, additionally ‘motivation’ was noted to be influenced by patients perceiving the benefits of the exercise, and adherence to the program was promoted by ‘goal-setting’. Other issues in the ‘capability’ domain included a fear and/or dislike of exercise.

**Conclusion:**

Patient engagement behavior has been shown to be influenced by both external (opportunity) and intrapersonal variables (capability and motivation). Those prescribing exercises within a rehabilitation program need to discuss these factors with their patients and co-design the exercise rehabilitation program in partnership with the patient, since this is likely to improve patient engagement, and thereby result in superior health outcomes. Furthermore, these factors need to be a consideration in clinical trials, if the findings from such trials are to translate into mainstream healthcare settings.

**Supplementary Information:**

The online version contains supplementary material available at 10.1186/s13102-022-00407-3.

## Background

Prescribed exercise is a fundamental component in the rehabilitation and treatment of numerous health conditions. Within these contexts it can be prescribed to target specific physical, metabolic, and neurological issues, as well as being integral to promoting the long-term general health and wellbeing of the patient. Examples include the treatment, alleviation, and management of conditions such as: cardiovascular disease, diabetes, musculoskeletal injuries, neurological conditions, and some cancers, as well as recovery from surgery [1–5]. However, despite the reported benefits of prescribed exercise for each of these, the rates of compliance to such programs have been reported to be less than 35% and can be less than 10% [6]. This lack of compliance with exercise rehabilitation programs means that optimal health outcomes may not be achieved by many patients: a scenario that affects not only the individual patient, but also their carers and support services. Hence, for the exercise prescriber, there is a need to understand the potential facilitators and barriers to patient engagement with a prescribed exercise program, and at an individual patient level utilise this understanding in order to promote adherence and thereby benefit the patient, other stakeholders and the healthcare system in general.

Commencing and then maintaining adherence to a prescribed exercise program requires behaviour change. The extent to which a patient is willing and able to make these behavioural changes is influenced by their motivation to engage with the exercise program as well as other facilitators and barriers that may be internal or external to the patient [7]. With some being within their control, whilst others are not. These factors, such as, self-efficacy, outcome-expectancies, risk perception, beliefs, etc., are recognised as personal factors of the World Health Organisation’s Classification of Functioning, Disability and Health (ICF) [8]. With researchers highlighting the importance of these factors in the field of rehabilitation [9, 10]. Of key importance in this respect is the patient’s psychological condition, which will affect their motivation, and will thereby be a significant engagement factor for all patients, regardless of any diagnosed clinical mental health condition.

To date, most current research into patient engagement relates to medication adherence and similar health-related behaviours. Whilst in the field of exercise rehabilitation there is a relative lack of clarity regarding patient adherent behaviours and the determinants of engagement with their prescribed programs. Developing an understanding of these is vital for healthcare practitioners prescribing exercise, so that they may be factored into the design of individual exercise programs for improved patient engagement. Furthermore, the patient’s circumstances are likely to change throughout their program, as for example, they may return to work causing some of their facilitators and barriers to engagement to alter. Thus, the considerations of each patient’s facilitators and barriers to engagement with their exercise program requires ongoing review as prescribed exercise programmes tend to extend over a prolonged period of time, and then require continued adherence post-rehabilitation for maintaining health improvements and preventing disease relapse.

The importance of applying an understanding of the factors affecting patient engagement are evident from the discrepancies between the benefits of exercise programs reported from clinical trials as compared to those attained when such programs are delivered in mainstream healthcare settings [11]. Several reasons have been proposed as causes of these discrepancies. Firstly, in the pursuit of optimal treatment outcomes, clinical exercise trials often invest a level of resources, such as staff time, facilities and other support, that can facilitate patient engagement and overcome key barriers to patient adherence. Unfortunately, this investment of resources and services may not be reflective of real-world healthcare settings. Secondly, the volunteers for clinical trials are likely to have a vested interest in the exercise activity, a belief in its efficacy, and be positively motivated to engage, as well as the time and resources to commit to the programme/clinical trial—characteristics that are not universal in the wider patient population. Consequently, treatment outcomes in the healthcare system are often of lesser magnitude than those demonstrated in clinical trials. Hence, exercise rehabilitation programs that are based solely on an idealised exercise prescription in idealised circumstances, without consideration of the factors that enable patient engagement, are likely to fail to achieve the optimal outcomes for many patients.

The aims of this review were therefore to identify the facilitators and barriers to patient engagement in exercise rehabilitation. The purpose was to identify these factors and thereby to highlight the need for their consideration in: (1) the decision making of clinicians when tailoring exercise rehabilitation programs to the specific circumstances and individual characteristics of their patients, so as to maximise engagement and through doing so, optimise health outcomes, and (2) the design of clinical exercise trials.

### The COM-B framework

Despite there being numerous theories on behaviour change, there remains a lack of consensus regarding a standardised approach to effectively analysing and characterizing the factors affecting behaviour change in health interventions. To address this, Mitchie et al. developed the COM-B (capability, opportunity, motivation—behaviour) behaviour change wheel to consider the sources of behaviour, intervention functions and impact of policies [12]. Within this interactive system, the three core conditions contributing to behaviour are; capability—consisting of both the physical and psychological capacity to engage in behaviour; opportunity—encompassing external physical and social environment factors; and motivation—categorized as the internal reflective and automatic processes that guide and direct behaviour. Since its publication the COM-B framework has been used in healthcare studies on diverse topics such as adherence to medication and practices preventing the spread of COVID-19﻿ [13, 14]. Given its efficacy, the COM-B framework was selected to be used in this review for the identification of the factors that influence patient engagement behaviour in exercise rehabilitation and its inferences for subsequent long-term maintenance of health benefitting physical activity.

## Methods

### Search strategy

**Fig. 1:**
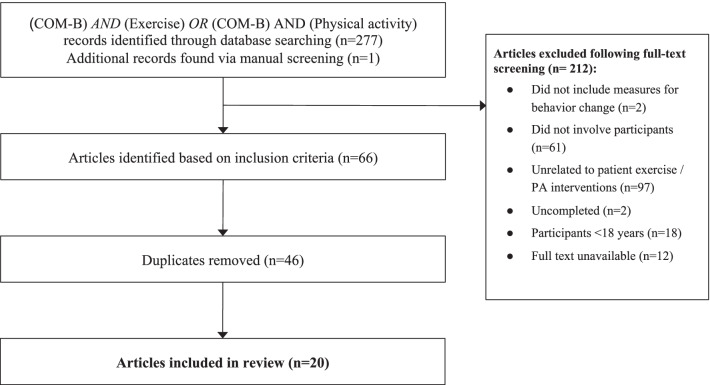
PRISMA diagram outlining search process

Referring to Fig. 1, the scoping review was conducted following Preferred Reporting Items for Systematic Reviews and Meta- Analysis (PRISMA) guidelines. Five electronic databases (PubMed, Cochrane, Embase, Web of Science, and ClinicalTrials.gov) were searched from inception until July 2021. The search terms included were ‘physical activity’, ‘exercise’ and ‘COM-B’. ‘COM-B’ was the specific keyword searched for within titles and abstracts, combined with either ‘physical activity’ OR ‘exercise’ included using the ‘AND’ operation. The search was limited to studies conducted in adult populations and availability in English.

### Selection criteria

The inclusion criteria were kept broad to ensure studies pertaining to measuring exercise or physical activity adherence were identified. However, articles must have included:

- Implementation of the COM-B framework for research with participants consisting of patients/clinicians involved in PA/exercise interventions for health conditions;
- Outcome measurements for behavioural analysis;
- Targeted at adult populations;
- Availability in English.

Full-text articles were then screened for relevance. Excluded articles were studies and trials that did not involve patient adherence to exercise, theoretical studies that did not involve participants, framework validity studies, protocols for research that had not yet been completed, and studies that focused on child participants.

### Data extraction, synthesis and analysis

Study characteristics such as study type, methods used, target population and health conditions, were extracted from the reviewed articles. Patient behavioural change variables were identified and extracted from the results and discussion sections of the reviewed articles and categorized according to their respective COM-B domains – Capability (physical and psychological), Opportunity (physical and social), and Motivation (reflective and automatic). Due to the lack of fixed definitions for the broad range of behavioural factors within the reviewed literature, extracted variables were categorised according to the guidelines of Michie et al*.* [12]. Additionally, the identified COM-B factors were classified as: Patient Facilitators (PF) defined as factors that increase exercise adherence; Patient Barriers (PB) defined as factors that decrease exercise adherence; Clinician Facilitators (CF) defined as factors that aid clinicians in increasing patient exercise adherence; and Clinician Barriers (CB) defined as factors that deter clinicians from increasing patient exercise adherence.

## Results

### Selection of sources of evidence

As shown in Fig. 1, 277 records were identified through database searching and one through manual searching. Two-hundred and twelve articles were excluded for not meeting the above-stated search criteria, and 46 duplicates were removed. Hence, twenty articles were included in this review. Nineteen articles included patient factors in their analysis, while only five studies had clinicians as research participants [15–19]. Five other studies that had not involved clinicians as participants had instead included recommendations for clinicians based on their research [20–24]. These recommendations were thereby extracted and mapped according to their respective COM-B domains.

### Study types

Referring to Table 1, studies were primarily qualitative and used semi-structured interviews. Out of the twenty papers reviewed; one study was on the evaluation of a randomised control trial using the COM-B framework [16]; one study involved the evaluation of a sustained lifestyle modification program [25]; ten studies were on behavioural analysis using the COM-B framework for health conditions [16, 17, 20, 21, 24, 26–30]; and eight were on developing a PA intervention based on patient and clinician responses to questions guided by the COM-B framework [18, 19, 22, 23, 31–34].

**Table 1:** Summary of characteristics of the included studies for the scoping review (n = 20)

Study	Type of health condition	Target population	Study type	Methods	COM-B factors identified^a^		
					CAP	OPP	MOT
Morris et al*.* [15]	Falls	Older adults prone to falls	Qualitative	Clinician interviews Patient focus groups	PF, PB, CF, CB	PF, PB, CF, CB	PF, PB, CF, CB
Ayton et al*.* [21]	Falls	Older adults prone to falls	Qualitative	Patient interviews Patient questionnaire	PF, PB, CF	PF, PB, CF	PF, PB, CF
Muhwava et al*.* [25]	Pregnancy	Mothers with gestational diabetes	Qualitative	Patient interviews Patient focus groups	PF, PB	PF, PB	PF, PB
Lucas et al*.* [16]	Pregnancy	Healthcare professionals supporting young mothers	Qualitative	Clinician interviews	CF, CB	CF, CB	-
Ellis et al*.* [26]	Pregnancy	Postnatal mothers	Qualitative	Patient interviews Patient questionnaire	PF	PF	PF
Flannery et al*.* [28]	Pregnancy	Overweight and obese pregnant women	Qualitative	Patient interviews	PF, PB	PF, PB	PF, PB
Handley et al*.* [33]	Pregnancy	Low income Latina mothers with gestational diabetes	Qualitative	Patient focus groups	PB	PB	PB
Ojo et al*.* [32]	Sedentary	Sedentary desk-based workers	Qualitative	Patient interviews	PF	PF	PF
Andersen et al*.* [20]	Sedentary	Patients prescribed with physical activity	Qualitative	Patient interviews	PF, PB, CF	PF, PB, CF	PF
Walker et al*.* [30]	Sedentary	British military veterans who are wounded/injured/sick	Qualitative	Patient interviews	PB	PB	PF, PB
Egerton et al*.* [17]	Musculoskeletal	Clinicians prescribing knee osteoarthritis management	Qualitative	Clinician interviews	CF, CB	CF, CB	CF
Govender et al*.* [27]	Cancer	Head and neck and cancer patients	Qualitative	Patient interviews	PF, PB	PF	PF, PB
Riemann-Lorenz et al*.* [24]	Neurological	Patients with multiple sclerosis	Quantitative Qualitative	Patient questionnaires	PF	-	PF
Silveira et al*.* [29]	Neurological	Patients with multiple sclerosis	Quantitative	Patient questionnaires	PF, PB	-	PF
Nyenhuis et al*.* [22]	Asthma	African American women impacted by both physical inactivity and asthma	Qualitative	Patient interviews Patient focus groups	PB, CF	PB, CF	PB, CF
Nyenhuis et al*.* [23]	Asthma	African American women impacted by both physical inactivity and asthma	Qualitative	Patient interviews Patient focus groups	PB, CF	PB, CF	PB, CF
Moore et al*.* [31]	Type II Diabetes	UK African and Caribbean communities with Type II Diabetes	Qualitative	Patient interviews Patient focus groups	PF, PB	PF, PB	PF, PB
Walsh et al*.* [18]	Cardiovascular disease	Patients with cardiovascular disease	Qualitative	Patient interviews Clinician interviews	PB	PF	PF, PB, CF
Hall et al*.* [19]	Cardiovascular disease	Stroke victims	Qualitative	Clinician and patient workshops	CF, CB	CF, CB	CF, CB
Levy et al*.* [34]	Cardiovascular disease	Stroke victims	Qualitative	Patient interviews	PF, PB	PF, PB	PF, PB

^a^PF, Patient Facilitator; PB, Patient Barrier; CF, Clinician Facilitator; CB, Clinician Barrier

### Participants

Referring to Table 2, a total of 2761 participants were included in the reviewed publications. Ninety-seven percent (n = 2676) of participants in the reviewed literature were ‘patients’, while only 3% (n = 85) were clinicians. Six studies involved only female ‘patients’, whereas one study was exclusively male. Seventy-five percent (n = 2014) of the ‘patients’ involved were female and 24.7% (n = 662) male. The age range of patients included adults aged from 16 to 90 years.

**Table 2:** Summary of participant characteristics (n = 2761)

Demographics	Patients	Clinicians
*Total participants*	2676 (96.9)	85 (3.1)
*Gender*		
Female	2014	35
Male	662	11
Unknown		39
Age range (years)	16–90	–*

*Not stated in research

### Health conditions

The types of health conditions and rehabilitation types included: fall prevention in older adults [15, 21], pregnancy-related conditions [16, 25, 26, 28, 33], musculoskeletal disease [17], physical inactivity [20, 30, 32], cancer [27], respiratory disease [22, 23], neurological disease [24, 29], type II diabetes [31], and cardiovascular disease [18, 19, 34].

### Facilitators and barriers

Table 3 summarizes clinician and patient behavioural determinants from the review articles. Behavioural determinants have been grouped according to their respective domains and further sub-categorized as facilitators and barriers to patient exercise adherence.

**Table 3:** Summary of patient and clinician behavioural determinants mapped to the COM-B framework

COM-B		Patient	Clinician
Capability	Facilitator	Knowledge about personal health [15, 21, 25, 32] Knowledge of suitable PA intervention strategies [15, 26, 28, 31, 32] Knowledge of PA guidelines [31, 32] Knowledge of the benefits of PA for their condition [12, 19, 32] Developing behavioural regulation skills (action planning and action control) [24, 27–29, 32, 34] Being fit prior to health condition [20, 24, 28] Physical capacity to engage in PA [15, 25, 27, 31, 32]	Providing patient with education to increase health knowledge Knowledge in client’s condition and condition management [15–17, 19] Experience with client’s condition and condition management [15, 17, 19] Communication skills (to facilitate lifestyle change) [16, 17, 19] Accounting for the patient’s previous experiences with PA when designing exercise programs [20] Tailoring PA to individual’s physical capacity [20]
Capability	Barrier	Limited knowledge about personal health [18, 25, 27, 30] Limited knowledge of suitable PA intervention strategies [21, 22, 26, 28, 30, 31] Limited knowledge of PA guidelines [26, 28, 31] Limited knowledge of PA benefits for their condition [21, 22, 27, 30] Negative perceptions (Fear/Dislike) of exercise [18, 20, 21, 23, 34] Perceived lack of time [21] Lacking behavioural regulation skills (action planning and action control) [22, 27, 28, 33] Poor mental health [30] Inadequate physical capacity to engage in PA [15, 18, 21–23, 25, 26, 30, 34] Pain/fatigue related to condition [27–30, 34]	Inadequate knowledge in client’s condition and condition management [15–17, 19] Lacking communication skills (to facilitate lifestyle change) [16, 17] Patient lacking strategies to regulate their own behaviour [19]
Opportunity	Facilitator	Easy access to PA resources and services [15, 18, 20, 25, 31, 32, 34] Affordable resources and services [15, 31] Adequate time for PA [15, 20, 25, 31, 34] Safe/suitable physical environment for PA [21, 25, 32, 34] Clinician support [15, 18, 25, 27, 34] Supportive primary healthcare provider [15, 34] Positive social/cultural influences [25, 31, 32] Social support (friends, family, partners) [18, 20, 25–28, 34] Social support (peers) [18, 23, 26, 28, 34]	Providing patients easy access to PA resources and services [17, 19, 20, 22] Participant’s perceived relevance [15] Incorporating exercise rehabilitation in a hospital/rehabilitation setting [21] Longer consultations to focus on PA [17, 19] Giving professional PA counselling and follow-ups [20] Issuing an exercise prescription to the patient [20] Providing educational material as basis for intervention [15] Supportive social influences/ enablers [16, 19] Using peer support groups for interventions [22, 23]
Opportunity	Barrier	Difficulty in accessing PA resources and services [20, 21, 28, 30, 34] Lack of time [15, 20, 21, 25, 26, 28, 30, 31, 33] Unable to afford resources and services [21, 26, 28, 30, 31] Unsafe/unsuitable physical environment for PA [22, 25, 26, 30, 33] Complex social situations [15, 30] Lack of social support [22, 23, 30, 33] PA not supported by patient’s primary healthcare provider [15] Unsupportive healthcare workers [25, 30] Social norms against exercise [30]	Patient’s competing priorities [15] Patient’s lack of perceived relevance [15, 16] Insufficient allocated time with patients [16, 17, 19] Lack of availability of resources for patients [17, 19] High costs to refer patients for exercise interventions [17] Negative social influences [17, 19]
Motivation	Facilitator	Perceived personal relevance [15, 18, 20, 21, 24–28, 30–32, 34] High intention for action [20, 24, 28, 29, 32, 34] Fear of consequences [28, 32] Sense of responsibility [25, 26, 28, 34] Self-efficacy [15, 18, 24, 28, 29, 31, 32] Enjoying doing PA [18, 20, 24, 26, 34] Effective use of goal setting [18, 24, 28, 29, 32, 34] Encouraging clinicians [15, 18, 20] Effective incentives to engage in target behaviour [15, 20, 27, 32, 34] Receiving emotional and mental support for condition [25, 28]	Patient-centred approach [15, 17, 18, 22] Providing health education to increase understanding of conditions [18, 21–23] Peer support [15, 22] Rapport with participant [15] Positive health messages (focus on positive aspects) [15, 21, 22] Providing constant encouragement [3] Clinician’s attitudes/behaviours during treatment (e.g. using optimistic tones towards rehabilitation treatment) [17, 19] Self-efficacy towards providing PA for patients with health conditions [19]
Motivation	Barrier	Lack of perceived personal relevance [15, 18, 21, 23, 26, 30, 31, 33, 34] Lack of self-efficacy [22, 23, 26, 28, 30] Emotional responses and mental issues related to condition [22, 25, 27, 28, 30, 34] Unable to break habits/mindsets [28, 30] Lack of enjoyment in doing PA [20, 30] In denial of condition [21]	Clinical decision-making within constraints of randomised control trial [15] Clinician’s attitudes/behaviours during treatment (e.g. using inconsistent tones towards rehabilitation treatment) [19] Improper use of goal-setting for patients [19] Lack of peer support [19]

#### Capability

The main themes identified for patient capability were knowledge, skills, and exercise perceptions. Types of health knowledge included: knowledge of personal health and health conditions [15, 21, 25, 30, 32], suitable PA intervention strategies [15, 22, 26, 28, 30–32], PA guidelines [31, 32], and benefits of PA for their well-being [15, 21, 22, 30, 32]. Two main forms of behavioural regulation skills were noted in several articles to influence behaviour, these being action-planning and action-control [22, 24, 27–29, 32, 34]. Action-planning was defined as being able to make detailed plans on how to complete target behaviour, while action-control was defined as the automaticity to control habits leading to target behaviour [24]. Moreover, these two skills were often grouped together and categorised as a single psychological capability factor for behavioural regulation in the articles. Contrarily, a lack of behavioural regulation skills resulted in lower levels of exercise adherence as patients forgot to do exercises, and/or only engaged in PA when they remembered to [27]. Ayton et al*.* [21] also noted that a perceived lack of time was a barrier to engaging in intervention programs, which may be attributed to a lack of behavioural regulation [20]. In addition, negative exercise perceptions, such as a dislike of exercise [18, 34] and a fear of exercise [18, 21], were noted to be psychological Capability barriers.

#### Opportunity

External themes identified were in the context of the patient’s physical and social environment. Multiple studies involving patients and clinicians identified a lack of access to resources and services as an opportunity barrier [20, 21, 28, 30, 34]. High costs of resources and services was also stated as a barrier for patients in five studies [21, 26, 28, 30, 31], whereas affordable facilities resulted in greater adherence levels [15, 31, 34]. A prominent physical opportunity determinant noted in multiple articles was the availability of time for engaging in PA [15, 20, 21, 25, 26, 28, 30, 31, 33]. Thus, inadequate time for PA is likely not limited to specific patient populations or demographics. A common reason stated ‘for lack of time’ was commitments to other priorities such as work and family [18, 20, 21, 25, 26, 28, 31]. In terms of social opportunity, family and friends were social facilitators in seven studies [18, 20, 25–28, 30], while five studies [18, 23, 26, 28, 30] had peer support as a facilitator. Conversely, a lack of social support opportunities was cited in four studies as a barrier to treatment adherence [22, 23, 33, 34].

#### Motivation

The main theme identified for Motivation was the development of autonomous patient motivation. Strong beliefs about capabilities (self-efficacy) were identified as strong facilitators developing autonomous motivation for patients in six studies [15, 18, 24, 29, 31, 32], whereas weak beliefs about capabilities was a motivational barrier in five studies [22, 23, 26, 28, 30]. Beliefs about social role and identity influenced beliefs about self-capabilities and had significant effects on exercise adherence [24, 28]. Two common facilitators of autonomous motivation were high intentions towards behavioural change [24, 28, 29, 32, 34], and the use of goal setting [18, 24, 28, 32, 34]. Other sources of motivation included: incentives to exercise such as rewards and positive health outcomes [15, 27, 32, 34], and enjoyment of exercise and physical activity [20, 24, 34]. In addition, twelve studies [15, 18, 20, 21, 24–28, 30–32, 34] found high perceived personal relevance to be associated with increased determination to engage in exercise behaviour. In contrast, nine studies [15, 18, 21, 23, 26, 30, 31, 33, 34] found patients with misconceptions about PA and their health conditions had lower exercise adherence. Common indications for high levels of perceived personal relevance included symptom control, and desire for better health and overall well-being.

#### Clinician strategies for facilitating patient adherence

In the context of Capability, three studies suggested the use of educational material by clinicians to increase their patients’ health literacy and increase exercise adherence [21–23]. Clinicians interviewed in three studies also placed emphasis on the importance of effective communication skills to relate information to patients and facilitate behaviour change [16, 17, 19]. It was further suggested that clinicians consider the patient’s prior experiences with physical activity during exercise prescription, as patients were less inclined to adhere to their programs when prescribed with unsuitable exercises [20].

Opportunity variables identified for clinicians were primarily extraneous and outside of the clinician’s control. These consisted of environmental variables such as: the patient’s life situation [15, 16], time allocated with the patient [16, 17, 19], patient access to intervention resources and services [17, 19, 20, 22, 23], and subjective social norms [16, 17, 19].

With regards to motivation, clinicians commonly stated the use of a patient-centred approach as a facilitator [15, 18, 22, 23]. Four studies [18, 21–23] cited the use of education, albeit focusing on making the information relevant to a patient for increasing patient perceived-relevance. In addition, two studies [15, 21] involving older adults found that patients responded more positively when the intervention details focused on positive aspects and health benefits.

## Discussion

A key barrier to patient engagement is their knowledge gap regarding personal health, their health condition, and the relevance of the prescribed exercise regimen, as misconceptions about health conditions and PA result in lower exercise adherence. This may be addressed through the provision of essential ‘educational’ information by the clinician in a way that emphasises its relevance to the patient and the positive outcomes they may attain through engagement with their exercise program. Furthermore, since patients reflected that they knowingly lacked the skills to develop the habits necessary for the intended behaviour [24, 27, 28, 32], it is apparent that simply prescribing the exercise is unlikely to result in high levels of adherence by the majority of patients. Hence, in addition to explaining the exercise regimen, clinicians need to invest time in strategies such as motivational interviewing, goal-setting, and discussing time management with their patients. Moreover, it is evident that the psychological condition of the patient is key in enabling behaviour change, and therefore psychological support, particularly in the case of chronic conditions, may facilitate greater engagement, as the patient becomes more capable of dealing with the consequences of their condition. Indeed, whilst there is little published research on the topic, there is a growing interest in combing ‘Acceptance and Commitment Therapy (ACT)’ with exercise in rehabilitation programs for chronic conditions [35]. A further benefit of this is that through working in this partnership the clinician and patient can develop a positive rapport, which has been reported to be one of the key motivational facilitators [15, 18, 20], as well as giving the patient ‘ownership’ of the program and thereby elevating their ‘self-efficacy’ and developing their autonomous motivation. The importance of this ‘shared decision making’ and inclusion of the patient’s personal preferences has previously been highlighted as a key aspect of facilitating adherence to a rehabilitation program in a study with osteoarthritis patients [36].

However, while high intentions for action have been highlighted in several psychological theories as a crucial predictor of engagement in PA, the fulfilment of intentions requires an enabling combination of other variables [37, 38], these being the presence of key facilitators and overcoming prohibitive barriers to the intended behaviour. Such factors include the patient’s background, demographics and life situations. Since, unlike participants involved in clinical research trials who may receive enabling access to optimal resources and services, members of the general population commonly face multiple environmental barriers to their engagement in rehabilitation, such as: a lack of access to resources and services as an opportunity barrier [20, 21, 28, 30], high costs of resources and service [21, 26, 28, 30, 31], and a ‘lack of time’ due to other various other personal commitments [18, 20, 21, 25, 26, 28, 31]. Hence if these factors are not considered in the patient’s exercise program they can affect opportunity, capability and motivation.

Addressing such barriers may inevitably result in some deviation from an idealistic exercise program for some patients. However, it is suggested that such compromises are likely to result in improved patient engagement that would result in superior health outcomes and adherence when compared with those that would result from an idealistic program that the patient fails to engage with. For example, whilst gym-based, one to one personal trainer sessions, several times a week may be ideal, if these are not feasible, alternative home-based exercise sessions that are more accessible, require no travel time, enable ongoing caring responsibilities and are zero cost, would be superior to no exercise. Related to this, when designing the program, the issues of ‘Realistic’ and ‘Attainable’ are essential aspects of any goal setting [18]. Hence for example, an idealised goal of five gym-based sessions a week may need to be adjusted to a more realistic number that the patient is capable of undertaking, since unattainable and unrealistic goals will be demotivating. Additionally, one strategy for overcoming some barriers and enabling greater engagement would be to incorporate the required exercises into the patient’s Activities of Daily Living (ADL’s) [39]: and whilst this may not be suitable for the inclusion of ‘specific exercises’, it may enable the inclusion of more general aspects of physical activity, should that be a goal of the program.

### Research implications

Referring to Table 4, to enable effective patient engagement in exercise rehabilitation, clinicians prescribing exercise should consider those factors under capability, opportunity and motivation in the design of each patient’s exercise program, and likewise for those designing clinical exercise trials.

**Table 4:** Research implications for improving patient engagement

COM-B	Recommendations for clinical decision-making/clinical trial designs
Capability	Include patient education—to inform the patient of the relevance of the exercise and how it will benefit them, also to ensure that they understand their condition and are not ‘in denial’ If the patient is required to complete sessions unsupervised, ensure that the patient has a clear guide of how to undertake their exercise sessions. This may include digital or hardcopy descriptions of their exercises, including factors such as duration, sets, repetitions and frequency of sessions. Where possible video clips that lead the patient through the session may be useful Dispel fears of the risk of injury and/or adverse events when participating in the prescribed exercise Identify the modes of exercise that the patient will undertake and if possible avoid those that they dislike
Opportunity	Ensure that the exercise sessions are affordable and accessible Ensure that the patient has time to undertake the exercise, including and travel time to locations Prescribe a program for which compliance will not be diminished by other priorities such as work, family and other commitments Where possible engage support structures and networks, such as friends and family
Motivation	Foster a positive relationship between the patient, their exercise prescribing clinician, and other health professionals they are working with Use motivational strategies, such as motivational interviews and goal setting If setting goals, ensure that they are desired, realistic and attainable for that patient, to reduce the risk of demotivation Work in partnership with the patient in the design of their program and goal setting to enhance their autonomous motivation, and program ownership Review the program design and associated goals regularly, not only in the context of the exercises that they are functionally capable of, but also in the context of their evolving Capabilities, Opportunities, and Motivations

### Limitations

Whereas multiple patient behavioural determinants were identified, there was limited data for informing clinician strategies for improving patient adherence within the scope of the review, since most studies had primarily focused on patient facilitators and barriers, rather than clinician factors. Moreover, there was a lack of clinician perspectives on behavioural determinants to patient engagement.

Additionally, while various behavioural determinants have been identified in the literature, none of the reviewed studies had compared differences in behavioural determinants between different types of health conditions. Some studies had patients with co- morbidities, such as falls prevention in older adults with musculoskeletal, cardiovascular, and metabolic disease [17], however the research outcomes were focused on the overall patient behavioural determinants toward a specific rehabilitation program (falls prevention), rather than comparing between different types of health conditions (differences in behavioural determinants between musculoskeletal, cardiovascular, and metabolic patients). Thus, not all the findings from this review may apply to all types of health conditions. Further studies should include additional analysis to investigate the differences between health conditions.

Lastly, there was a lack of data for certain types of health conditions. The majority of participants were patients with a neurological disease (n = 1881). On the other hand, cancer (n = 13) and musculoskeletal (n = 11) patients had the smallest samples sizes, contributing 0.5% and 0.4% to the total participant sample size respectively. Moreover, no studies were identified in which the COM-B model had been used to assess the facilitators and barriers to exercise engagement for participants with clinical mental health conditions. This is a key deficit since as previously indicated, the psychological state of the participant is vital in influencing their motivation to engage with the exercise program in all scenarios, including those in which the patient may otherwise be regarded as have good mental well-being, but will have further implications if they have a clinical mental health condition.

## Conclusion

Improving patient engagement in exercise rehabilitation requires behaviour change. This scoping review investigated previous studies using the COM-B model in exercise rehabilitation to identify behavioural determinants of patient adherent behaviour. Various intrapersonal (Capability and Motivation) and external (Opportunity) determinants were identified and shown to be relevant when designing interventions.. It is evident that in addition to Capability and Opportunity factors presenting as potential facilitators and barriers, they also have the potential to be a significant influence on patient Motivation: an attribute that is also affected by the general mental and psychological state of the participant. Ultimately, individual exercise rehabilitation programs should be co-designed between the patient and exercise specialist, and in this process the engagement factors given due consideration, in order to optimise participation and thereby attain the best outcomes for the participant. The relative influence of these factors will differ between individuals and may also portray a greater or lesser influence with different health conditions. Future research should consider including additional outcome measures to determine the significance of these factors for a greater understanding of patient engagement.

## Supplementary Information

**Additional file 1**. Lay-person summary.

## Data Availability

All data generated or analysed during this study are included in this published article, its Additional file 1 and the publications referenced in this manuscript. Original data files and analyses are stored in accordance with the authors’ institution’s data storage and management policy, and are available upon request.

## Abbreviations

CB: Clinician barriers
CF: Clinician facilitators
COM-B: Capability, opportunity, motivation-behaviour
PRISMA: Preferred reporting items for systematic reviews and meta-analysis
PA: Physical activity
PB: Patient barriers
PF: Patient facilitators
