# Supplementary material for: Identifying the factors affecting ‘patient engagement’ in exercise rehabilitation
Source: BMC Sports Sci Med Rehabil. 2022 Feb 7;14:18. doi: 10.1186/s13102-022-00407-3 (PMC8819209; doi:10.1186/s13102-022-00407-3)
Supplement: Supplementary file 1 — Additional file 1. Lay-person summary. [file 13102_2022_407_MOESM1_ESM.docx]

**Identifying the factors affecting ‘Patient Engagement’ in exercise rehabilitation**

Junsheng L Teo ᵃ, Zhen Zheng ᵃ, Stephen R Bird ᵃ*

^a^ School of Health and Biomedical Sciences, RMIT University, Melbourne, Australia, 3083

* Senior and corresponding author (stephen.bird@rmit.edu.au)

**Lay language summary**

It is well established that prescribed exercise can be extremely beneficial in helping patients to recover from injury and illness, as well as providing general health benefits. These benefits include: the gradual strengthening of muscles, tendons and joints; promoting good posture and effective use (recruitment) of the correct muscles in movement; and numerous other health improvements. Hence, many patients are referred to exercise specialists (exercise physiologists, physiotherapists and others) for the prescription of exercise programs to aid their recovery and promote their general health. However, despite accessing these services, relatively few patients fully adhere to their prescribed exercise program. This means that many patients fail to gain the full benefits of the prescribed exercises, and consequently may not improve or recover as quickly, effectively, or fully as they might. This failure to achieve optimal health and/or recovery can thereby have adverse consequences for the patients and others who support them.

It is inevitable that patients have other life commitments and priorities, and that these may in some ways conflict with and prevent them from completing their exercises as fully or as often as prescribed. Exercise specialists therefore need to prescribe the patient’s exercise program in the context of these other ‘life’ and personal factors. Through understanding the situation of the patient, the factors that may prevent them from exercising, and the factors that may encourage/enable them to exercise, the exercise specialist can then consider these in the prescription of the patient’s exercise program, and in doing so, enable greater compliance. Based on our research findings we recommend that those prescribing exercise need not only to advocate an exercise program that includes effective: types of exercise, undertaken at the optimal intensity, frequency and duration, but where necessary to refine the program in accordance with the factors that are most likely to enable the patient to comply with the prescribed exercise. These considerations are essential for effective exercise prescription and the relevant personal, social, environmental and lifestyle factors would need to be discussed with a patient. In doing so the development of the exercise program becomes a collaborative process in which the patient is likely to develop a sense of program ownership, improved self-efficacy, and as a result engage more fully with their program.

Examples of the factors that the exercise prescriber and patient need to consider include:

1. Ensuring that the patient understands how and why the exercises will promote their recovery and general health, as well as dispelling any fear the patient may have about the suggested exercise.
2. Confirming that the prescribed exercise regimen is realistic in terms of time commitments, and involves planning of when and where the exercises can realistically be undertaken, so that they can be easily incorporated into the patient’s lifestyle.
3. Where possible enabling the patient to get the social support and encouragement of others (if this is an issue).
4. Prescribing exercises that can be undertaken at locations that do not require prohibitive travel or financial costs – both of which may be significant barriers to patients with other important personal, social and family commitments.
5. Exercises that the patient is more likely to enjoy, this may include some social or environmental aspect, whereas a dislike of the exercise is likely to prevent participation.
6. Other processes such as ‘Goal setting’ may also be beneficial, as the patient may gain positive encouragement on a weekly basis, particularly when the overall program to recovery and optimal health may be over a prolonged duration.

It is recognised that refining an exercise program in accordance with the above factors may result in changes from a theoretically ideal program, but as previously indicated, such ‘optimal’ theoretical prescriptions are ineffective if a patient does not engage with the program. Whereas effective engagement with a ‘modified’ exercise program, that aligns with the patient’s aspirations, lifestyle and capabilities is likely to produce better rehabilitation, recovery and health outcomes.
